# Agency Nursing Staff Utilization and Turnover in Nursing Homes: A Longitudinal Analysis

**DOI:** 10.3390/healthcare13040379

**Published:** 2025-02-11

**Authors:** Rohit Pradhan, Ganisher Davlyatov, Latarsha Chisholm, Cynthia Williams, Keya Sen, Amelia Manning, Robert Weech-Maldonado

**Affiliations:** 1 School of Health Administration, Texas State University, San Marcos, TX 78666, USA; keyasen@txstate.edu; 2 Department of Health Administration and Policy, University of Oklahoma Health Sciences Center, Oklahoma City, OK 73104, USA; 3 School of Global Health Management and Informatics, University of Central Florida, Orlando, FL 32827, USA; latarsha.chisholm@ucf.edu (L.C.);; 4 St David’s School of Nursing, Texas State University, San Marcos, TX 78666, USA; 5 Department of Health Services Administration, University of Alabama at Birmingham, Birmingham, AL 35294, USA

**Keywords:** nursing homes, agency nursing staff, contract nursing staff, turnover

## Abstract

**Background/Objectives**: Nursing staff turnover can adversely affect nursing home (NH) performance. To address staffing shortages, NHs are increasingly turning to agency nursing staff as a solution. This study examined the relationship between the use of agency nursing staff and turnover rates among NH permanent nursing staff. **Methods**: This retrospective, observational study used secondary data from several sources, including the Payroll-Based Journal, the Care Compare: Five Star Quality Rating System, and Area Health Resource Files (n: =35,200, years: 2021–2023). The dependent variable was turnover rates among registered nurses (RNs), licensed practical nurses (LPNs), and certified nursing assistants (CNAs). The independent variable was the classification of NHs based on their level of agency nursing staff utilization. Facilities were classified as “high utilizers” (the top 25% in agency nursing staff use) and “low utilizers” (the remaining 75%). This classification was informed by prior research indicating that the impact of agency nursing staff on NH performance is most pronounced at higher levels of utilization. A two-way fixed-effects regression model (facility and year) was used, with appropriate control variables. **Results:** NHs identified as high utilizers had significantly higher turnover rates among permanent RNs (7%) and CNAs (1.9%) compared to facilities that had low utilization of agency nurses (*p* < 0.001). No significant association was found between agency LPN utilization and LPN turnover. **Conclusions:** Greater reliance on agency nursing staff was associated with increased turnover, with the strongest effect observed for RNs. NH administrators should consider strategies to balance agency staff utilization with efforts to retain permanent staff, emphasizing long-term workforce stability.

## 1. Introduction

Nursing staff, including registered nurses (RNs), licensed practical nurses (LPNs), and certified nursing assistants (CNAs) are the primary caregivers in nursing homes and are critical to delivering high quality care [1]. However, high turnover rates among nursing staff have been a long-standing concern in nursing homes [2]. A recent study using data from the national-level Payroll-Based Journal (PBJ) reported alarmingly high median turnover rates of 102.9% for RNs, 79.8% for LPNs, and 98.8% for CNAs [3]. Although turnover rates reported in other recent studies vary [4,5]—largely due to inconsistent definitions of turnover—nursing staff turnover remains a salient issue that demands attention.

Nursing staff turnover can adversely affect nursing home performance. For instance, turnover has been associated with a higher number of quality of care deficiencies and increased mortality among nursing home residents [6,7]. A study by Zheng and colleagues [5] has reported that nursing staff turnover may be associated with poorer quality outcomes such as increased emergency department visits and a reduced likelihood of achieving higher star ratings in the Care-Compare: Five-Star Quality Rating System (Five-Star QRS).

Furthermore, increased turnover can also be financially burdensome for nursing homes due to the significant expenses associated with recruiting, hiring, and training new employees. These costs can include advertising job openings, conducting interviews, onboarding, and dedicating time and resources to training [4]. Such expenses can divert resources away from other critical investments, such as improving resident services and maintaining overall care quality [8].

Prior research has linked nursing staff turnover to various factors such as job design [9], facility characteristics such as quality [3,10], leadership turnover [11], organizational commitment [12], job satisfaction [13], and wages [4]. However, little is known about how nursing staff patterns, such as the use of agency labor, influence turnover.

Agency or contract staff are temporary professionals employed by third-party agencies to fill staffing gaps across various facilities [14]. In recent years, the use of agency nursing staff has increased significantly in nursing homes, particularly due to staffing shortages precipitated by the COVID-19 pandemic. For instance, in 2018, 23% of nursing homes used agency nursing staff, accounting for 3% of direct care hours. By 2022, nearly half of nursing homes used agency staff, accounting for 11% of direct care hours [15]. Hiring agency nursing staff offers certain advantages such as flexible scheduling, reduced overtime for regular staff, and decreased need to create permanent positions [16]. Agency nursing staff can also shield nursing homes from sudden shortages due to absenteeism or a surge of residents [17].

Despite these benefits, the utilization of agency nursing staff raises concerns about its potential impact on nursing home performance. While prior research has explored associations between agency nursing staff utilization and quality [18,19] as well as the ownership factors influencing their use [17], there is limited understanding of how agency staff utilization influences turnover among permanent staff. This study addresses this gap by investigating the relationship between agency nursing staff utilization and the turnover rates among permanent nursing staff in nursing homes.

Given the acute shortage of nurses in the United States (U.S.) healthcare system, reliance on agency labor is likely to persist for the foreseeable future. Therefore, it is crucial to understand how agency labor affects key nursing home performance metrics, such as turnover. By highlighting these important dynamics, the findings of this study may help policymakers and nursing home administrators better understand the implications of agency nursing staff utilization on nursing home performance. Ultimately, the findings could inform strategies to sustain high-quality care while addressing the challenges of workforce retention.

## 2. Methods and Materials

This study was a retrospective observational analysis that included all Centers for Medicare and Medicaid Services (CMS) certified nursing homes in the U.S. The analysis utilized secondary data from publicly available datasets maintained by U.S. federal agencies. These datasets are collected and updated regularly to ensure accurate and reliable reporting of nursing home characteristics, staffing, and quality metrics. The study period covered 2021–2023, during which a total of 35,200 facilities were analyzed. This study followed STROBE reporting guidelines for observational studies to ensure transparency and rigor in its methodology.

### 2.1. Data Sources

The study utilized several publicly available secondary datasets maintained by federal agencies and research organizations in the U.S. The PBJ dataset is managed by CMS and includes detailed staffing data reported quarterly by all CMS certified nursing homes [20]. Facilities are required to submit staffing information through this system to comply with federal regulations. The PBJ data were used to calculate the turnover variables. Also maintained by CMS, Five-Star QRS assesses nursing home quality using a combination of staffing, health inspections, and quality measures [21]. The data are updated monthly based on facility reports and CMS audits. The Provider of Service File (POS) [22], maintained by CMS, and Area Health Resource Files (AHRF) [23] overseen by the Health Resources and Services Administration were used to calculate nursing home organizational and market-level variables. The Post-Acute Care and Hospice Provider Utilization and Payment Public Use Files [24], published annually by CMS, were used to calculate the hierarchical condition category (HCC) risk score, while the Robert Graham Center data captured the Social Deprivation Index (SDI) [25]. A complete list of variables, along with their definitions and data sources, is provided in Supplementary Table S1.

The initial PBJ dataset contained 45,133 observations. It was first merged with the Five-Star QRS data, resulting in 44,750 observations, with 383 unmatched records. Next, merging with the POS data reduced the total to 44,711, leaving 39 unmatched records. The subsequent merge with PAC-PUF led to a decrease to 37,378 observations, as 7333 records were unmatched. Integrating the AHRF and SDI did not cause additional data loss, maintaining 37,378 observations. The CMS Certification Number (CCN) was used for merging, except for the SDI, which utilized the Federal Information Processing Standards (FIPS) code. After removing 2178 records having missing variables necessary for analysis, the final analytical sample comprised 35,200 nursing homes, representing an average of 11,733 unique facilities per year over the 2021–2023 study period.

### 2.2. Variables

#### 2.2.1. Dependent Variable

The dependent variables consisted of the turnover rates for permanent RNs, LPNs, and CNAs. While licensed nurses (RNs and LPNs) manage care plans and supervise other staff, nurse aides (CNAs) are predominantly responsible for providing direct resident care [1]. The turnover rate was calculated by dividing the number of staff who left during the year by the average number of staff employed. For example, if 10 RNs left a facility during the year and the average number of RNs employed that same year was 100, the RN turnover rate would be 10%. Only permanently employed nursing staff who were involved in resident care were included [15].

#### 2.2.2. Independent Variable

The independent variable was the classification of nursing homes based on their level of agency nursing staff utilization. Facilities in the top 25% of agency nursing staff use were classified as high utilizers while the remaining 75% were categorized as not-high utilizers. This dichotomization was based on the proportions of agency labor for RNs, LPNs, and CNAs, calculated as $(\frac{agency\;nursing\;staff\;hours}{total\;nursing\;staff\;hours})\;*\;100$ for each type of nursing staff at the facility level. These calculations were based on the PBJ data, which offers a daily record of the number of hours worked by each category of nursing staff (RNs, LPNs, and CNAs). Research has shown that the use of agency nursing staff has the greatest negative impact on quality at the highest levels of utilization [26]. Therefore, the dichotomization allowed for an examination of whether the facilities with the greatest reliance on agency nursing staff experienced a more substantial impact on turnover compared to those with lower levels of agency staff utilization.

#### 2.2.3. Control Variables

We controlled for facility-level and community-level characteristics of a nursing home that may affect the relationship between agency nursing staff and turnover [4,5,17]. The facility-level control variables included the following: size (number of certified beds), occupancy rate (percentage of occupied beds), nursing staff (RN, LPN, and CNA) hours per resident day (PRD), hierarchical condition category (HCC) risk score to reflect resident acuity, and quality star rating (1–5 values, with higher value indicating better quality).

The community-level control variables were measured at the county level and included SDI (a composite measure of socioeconomic factors such as poverty, education, and employment), full-time nursing staff employment in hospitals (the total number of full-time nursing staff (RNs, LPNs, and CNAs) employed in hospitals per 1000 population), market competition, and Medicare Advantage (MA) penetration (the percentage of Medicare beneficiaries enrolled in MA). Market competition was assessed using the Herfindahl–Hirschman Index (HHI)–a metric that evaluates market concentration by summing the squared market shares of each nursing home based on beds. The HHI ranges from 0 to 1, where higher values indicate a higher concentration of market power, signaling less competition, while lower values reflect a lower concentration and greater competition among market participants [27].

Certain variables such as ownership (for-profit, not-for-profit, government), chain affiliation (yes, no), and location were excluded from the models because facility-level fixed effects inherently account for time-invariant characteristics, making these variables redundant.

### 2.3. Analysis

The unit of analysis was the nursing home. The study employed a retrospective observational design using secondary data. We used descriptive statistics to summarize our dependent, independent, and control variables using means and standard deviations (SDs) for continuous variables and frequencies and percentages for categorical variables.

We modeled the data using linear regressions with two-way fixed effects at the facility and year levels. Facility-level fixed effects controlled for unobservable, facility-specific characteristics that remained constant over time, such as human resource practices or culture, to ensure that our analysis isolated the impact of the variable of interest (agency labor) on nursing staff turnover. It also reduced the risk of omitted variable bias [28]. Year fixed effects control for time trends. Separate models were run for RNs, LPNs, and CNAs. We found no evidence of multicollinearity among the variables (i.e., Variance Inflation Factor (VIF) ≤ 5, correlation coefficients (r) < 0.8). Stata 16.1 was used for the statistical analysis. Statistical significance was evaluated at an alpha level of 0.05 or less.

## 3. Results

The final analytic data file comprised 35,200 nursing homes with an average of 11,733 unique facilities per year. Table 1 provides descriptive statistics summarizing the key variables and highlights significant changes between 2021 and 2023. Turnover rates increased significantly across all nursing staff types. For RNs, turnover rose from 63.1% to 87.7%, for LPNs from 42.4% to 64.6%, and for CNAs from 49.1% to 67.1% (*p* < 0.001). The proportion of agency nursing staff hours PRD increased significantly: for RNs, it rose from 6.2% to 8.9%, for LPNs from 8.9% to 11.6%, and for CNAs from 7.4% to 9.3% (*p* < 0.001). Nursing hours PRD decreased slightly for RNs and LPNs but remained stable for CNAs, with only the changes for RNs and LPNs being significant (*p* < 0.001). Quality ratings also shifted significantly, with an increase in facilities rated with one or two stars and a decrease in those rated five stars (*p* < 0.001).

The fixed-effects regression analysis showed that high utilizers of agency labor experienced significantly higher nursing staff turnover rates (Table 2). High agency utilizers had approximately a 7.7% higher turnover compared to low utilizers among RNs (*p* < 0.001). Similarly, high utilizers of agency CNAs experienced a 1.9% higher turnover among permanent CNAs (*p* < 0.05). However, the analysis found no significant impact of high agency LPN utilization on LPN turnover.

In addition to agency staff utilization, several covariates were examined for their association with turnover rates. Higher occupancy rates and increased nursing staff hours PRD were consistently associated with lower turnover across all staff types (*p* < 0.001). Additionally, higher quality (star ratings) corresponded to reduced LPN turnover, with five-star facilities showing approximately a 9.1% lower turnover (*p* < 0.001). In contrast, a higher Medicare Advantage rate was linked to a 1.2% higher CNA turnover rate (*p* < 0.001).

## 4. Discussion

Nursing staff are critical to nursing home performance, and high turnover rates have been a long-standing concern in the industry. As nursing homes increasingly turn to agency nursing staff to address staffing gaps, understanding the implications of these staffing dynamics is essential. This study aimed to evaluate the relationship between the use of agency nursing staff utilization and turnover rates in nursing homes. It compared facilities having high reliance on temporary nursing staff with those having lower reliance to determine if greater utilization leads to increased staffing instability.

In this study, the relationship between agency nursing staff and turnover appeared to be nuanced, having varying effects across different types of nursing staff. The findings suggest that the most significant influence was observed among RNs, where higher utilization of agency RNs was significantly associated with increased turnover. In contrast, the impact on CNAs was smaller, while no significant relationship was found for LPNs.

Broadly speaking, there are many reasons why agency nursing staff may be associated with higher turnover. As agency nursing staff may be unfamiliar with the facility’s protocols, residents’ specific needs, and the organizational culture [19], they can create an additional workload for the permanent staff [29]. The permanent staff may need to assist or supervise agency labor, leading to increased stress and job dissatisfaction. A survey conducted among nursing home administrators revealed that increased supervision is one of the most frequently reported disadvantages of using agency nursing staff [29]. This added responsibility can contribute to burnout and a desire to leave the organization [13].

Furthermore, an overt reliance on agency nursing staff may signal to the permanent staff that the facility is not invested in long-term staffing solutions. This perception can diminish morale and organizational commitment among permanent staff, factors previously associated with turnover [12]. Moreover, agency labor can hinder the development of strong team dynamics and trust among staff members. This may be particularly acute when agency labor becomes a semi-permanent substitute for directly employed labor, as is increasingly apparent in some nursing homes [15].

Additionally, agency labor is significantly more expensive than regular nursing staff; for instance, in 2022, agency RNs in nursing homes cost almost $24 more per hour than directly employed RNs [30]. These significant salary differentials may fuel discontent among the permanent staff, leading to higher turnover. Equity theory suggests that employees assess fairness by comparing their input–output ratios to those of their peers; perceived inequities can lead to decreased job satisfaction and increased turnover intentions [31]. Permanent nursing staff may believe that the resources devoted to hiring agency labor could have been invested in retention strategies, such as competitive salaries, benefits, or professional development opportunities. Finally, the use of agency nursing staff has been linked with lower quality in nursing homes [18], which can be demoralizing for permanent staff committed to providing high-quality care. Research suggests that lower quality nursing homes may experience higher turnover [3].

Turning to the specific findings of this study, the most pronounced impact was observed among the RNs, where higher reliance on agency RNs strongly correlated with increased RN turnover. RNs are the cornerstone of nursing home quality and hold critical roles in resident care coordination, clinical decision-making, infection prevention and control, and leadership within the care team [1]. Introducing agency RNs, who may be less familiar with the facility’s protocols and resident-specific care plans, may lead to inconsistencies in care delivery. Permanent RNs may experience increased workloads as they assist agency RNs in acclimating to the facility’s environment, which can lead to job dissatisfaction and burnout.

Moreover, RNs possess advanced skills and qualifications that make them highly sought after across various healthcare settings, especially given their acute shortage in the U.S. healthcare system [32]. For instance, hospitals typically offer higher salaries, better benefits, and more opportunities for professional development compared to nursing homes [33,34]. The higher job mobility means that when permanent RNs face disruptions in their work environment due to high use of contractual labor, they may be more inclined to seek employment elsewhere. Furthermore, the need to frequently integrate agency RNs might hinder the development of cohesive teams, affecting communication and trust among staff members and further motivating permanent RNs to seek outside opportunities.

In contrast, the utilization of agency CNAs appeared to exert a relatively modest impact on turnover among permanent CNAs. CNAs are primarily responsible for providing direct resident care, assisting with the activities of daily living, and addressing the needs of cognitively challenged residents [1,35]. Given that CNAs already experience high baseline turnover rates in nursing homes [3], the additional impact of agency CNAs may be less material. While the use of agency CNAs may introduce some challenges, permanent CNAs might be more adaptable to working alongside agency staff due to the task-oriented nature of their roles [36]. However, the modest increase in turnover indicates that some level of dissatisfaction or strain exists, possibly due to inconsistencies in care approaches or increased responsibilities in guiding agency CNAs.

Finally, the absence of a significant relationship between agency LPN utilization and LPN turnover is an interesting finding that warrants further research. LPNs often occupy a middle ground between RNs and CNAs, performing duties that include administering medications and monitoring resident conditions [1]. As LPNs operate under the supervision of RNs, the nature of their work may involve more standardized procedures, making it easier for agency LPNs to integrate within the care team without causing significant disruption. Additionally, other factors not captured in this study, such as job satisfaction levels or career advancement opportunities, may play a more significant role in influencing LPN turnover.

An interesting finding of this study was that increased nursing staff hours were consistently associated with lower turnover rates across all categories. Adequate staffing ratios can alleviate the workload on individual caregivers, reduce job-related stress, and enhance job satisfaction [37]. CMS has finalized new nursing home staffing requirements, including a minimum of 0.55 RN hours PRD [38]. While these regulations have faced legal challenges [39], our study findings indicate the importance of enhanced nursing staff hours in nursing homes, which may reduce turnover and, in turn, improve quality.

This study has several limitations. The observational design does not establish causality, and it is possible that higher turnover rates drive the increased use of agency nursing to fill staffing gaps, creating a feedback loop that complicates the interpretation of results. Although the facility-level fixed-effects model helps account for time-invariant characteristics or within-facility variation, it does not address the concern of reverse causality. Future researchers should consider employing more advanced techniques, such as instrumental variable approaches, to better address this issue and strengthen causal inferences. Second, the lack of consistent definition of turnover in literature may complicate comparisons across studies. Variability in how turnover is defined and measured can impact the generalizability of our findings and may contribute to differences observed in turnover rates reported by other studies. This inconsistency underscores the need for standardized definitions and measures of turnover in future research. Third, the data used in this analysis were not specifically collected to study turnover, which may limit the precision of the turnover variable. Missing data may also introduce biases, as facilities with incomplete data might differ systematically from those with complete records; however, given the large sample size, this is unlikely to be a significant concern. Fourth, confounding variables, such as leadership styles, job satisfaction, organizational culture, and work environment characteristics, were not captured or controlled for in this analysis. These unmeasured factors may have influenced both turnover rates and agency nursing staff utilization, potentially introducing bias. Future research should evaluate the wage gap between agency nursing staff and permanent staff to understand how it mediates turnover. Finally, we lack information on the tenure of nursing staff and the type of departure (voluntary or involuntary). This information is important to fully understand the dynamics of turnover in nursing homes. Thus, there is a need to conduct qualitative studies focused on understanding the specific reasons for staff separations in nursing homes.

### Policy and Practical Implications

The sharp growth of agency nurses in NHs has offered a seemingly convenient solution to address chronic nursing staff shortages. Our results underscore the potential pitfalls of this approach, as it may be associated with increased turnover among the nursing staff.

Efforts to reduce reliance on agency nurses by improving the recruitment and retention of permanent nurses could also be beneficial. However, addressing the structural issues behind the U.S. nursing shortage will require significant policy interventions and is unlikely to materialize in the short term. In the interim, agency nurses are a reality. Therefore, targeted interventions such as competency assessments and fostering collaborative opportunities that harness their potential while safeguarding nursing home performance are crucial. Policymakers can play a significant role by incentivizing stable staffing models through financial incentives, enacting stricter qualification and training standards for agency nursing staff, and ensuring adequate funding for long-term care workforce development [35]. Collaboration between nursing home leaders and policymakers is crucial to implement these strategies effectively and to promote a sustainable, high-quality workforce that meets resident needs.

These results have important implications for staffing strategies in nursing homes. High turnover among the nursing staff and the use of agency labor are inextricably linked; however, there is no single solution or one-size-fits-all approach to address this issue. To mitigate high rates of turnover among licensed nurses such as RNs, facilities should provide a supportive work environment that includes elements such as “the implementation of evidence-based practices; shared decision making regarding resident care, staffing, and the work environment, involvement, and leadership in quality improvement initiatives; and support for professional development” [35].

While strategies for licensed nurses should focus on creating a supportive professional environment, CNAs face different challenges that may require alternative approaches. Despite the arduous nature of their work, CNAs receive low wages and frequently lack benefits, resulting in poverty-level incomes [35,40]. Research suggests that improved CNA wages and the provision of health insurance benefits may be associated with reduced turnover [4,41]. Nursing homes should also consider providing additional training for CNAs including career advancement and professional development opportunities, as these are not only associated with higher quality [42] but also with higher job satisfaction [43]. Moreover, given that nurse aides are disproportionately women from minority backgrounds, it is important to address existing disparities related to institutional racism and sexism [44,45].

## 5. Conclusions

Nursing staff are crucial determinants of nursing home performance. Faced with long-standing workforce shortages, nursing homes have increasingly turned to agency labor to address staffing gaps. This study contributes to the literature by showing that increased use of agency nursing staff within nursing homes may be associated with higher turnover, particularly among RNs. Given that turnover can negatively impact nursing home performance, administrators should carefully balance the use of agency nursing staff to meet immediate needs against its potential negative impact on staff retention and overall performance. Implementing targeted strategies to improve the recruitment and retention of permanent staff, along with policy initiatives that incentivize stable staffing models tailored to the unique needs of nursing homes, may help reduce the reliance on agency nursing staff, reduce turnover, and ultimately improve resident care quality.

## Figures and Tables

**Table 1:** Descriptive statistics (N = 35,200).

	2021	2023	*p*-Value
Variable	Mean (SD)/Freq (%)	Mean (SD)/Freq (%)	
Turnover rate			
RN	63.11 (64.28)	87.71 (82.47)	<0.001
LPN	42.43 (43.47)	64.59 (67.28)	<0.001
CNA	49.11 (41.05)	67.08 (60.47)	<0.001
Agency nurse share			
RN	6.16 (12.54)	8.85 (15.47)	<0.001
LPN	8.18 (13.30)	11.61 (16.72)	<0.001
CNA	7.41 (11.69)	9.25 (13.71)	<0.001
Size (certified beds)	110.37 (59.79)	109.73 (58.42)	0.401
Occupancy rate	72.69 (16.16)	76.08 (16.26)	<0.001
Nursing staff hours PRD			
RN	0.45 (0.31)	0.42 (0.30)	<0.001
LPN	0.81 (0.31)	0.79 (0.31)	<0.001
CNA	2.002 (0.56)	2.02 (0.54)	0.056
HCC risk score	2.63 (0.66)	2.578 (0.621)	<0.001
Quality (star rating)			
One-Star	657 (5.5%)	877 (7.5%)	<0.001
Two-Star	1617 (13.6%)	1864 (16.0%)	
Three-Star	2579 (21.8%)	2521 (21.7%)	
Four-Star	3174 (26.8%)	3173 (27.3%)	
Five-Star	3829 (32.3%)	3186 (27.4%)	
Social Deprivation Index	50.98 (26.66)	50.914 (26.603)	0.851
FTE nursing staff employment in hospitals			
RN	2892 (5644)	2871 (5651)	0.778
LPN	121 (231)	112 (230)	0.701
CNA	718 (1368)	712 (1369)	0.737
Competition (HHI)	0.21 (0.24)	0.21 (0.25)	0.284
Medicare Advantage rate	41.40 (13.40)	44.39 (13.12)	<0.001

Notes: N: number of nursing homes, SD: standard deviation, Freq: frequency, RN: registered nurse, LPN: licensed practical nurse, CNA: certified nursing assistant, PRD: per resident day, HCC: hierarchical conditional category, FTE: full-time equivalent, HHI: Herfindahl–Hirschman Index.

**Table 2:** Fixed-effects regression analysis of the relationship between use of agency nursing staff and nursing staff turnover (N = 35,200).

Variable	RN Turnover	LPN Turnover	CNA Turnover
Agency nursing staff utilization (0: not high utilizers; 1: high utilizers)			
RN	7.702 ***		
LPN		0.580	
CNA			1.873 *
Size (certified beds)	0.117	−0.021	−0.006
Occupancy rate	−0.327 ***	−0.315 ***	−0.318 ***
Nursing staff hours per resident day			
RN	−68.057 ***		
LPN		−24.232 ***	
CNA			−12.169 ***
HCC risk score	0.724	−0.709	0.013
FTE nursing staff employment in hospitals			
RN	−0.013		
LPN		−0.144	
CNA			−0.038
Social Deprivation Index	−0.472	−1.130 *	−0.14
Quality star rating			
One-Star			
Two-Star	−1.144	−1.312	0.742
Three-Star	−3.378	−4.617 *	−0.598
Four-Star	−3.526	−7.200 ***	−1.918
Five-Star	−4.66	−9.107 ***	−3.529
Competition (HHI)	−28.549	−6.054	7.777
Medicare Advantage rate	0.892	−0.081	1.192 ***

Notes: * *p* < 0.05, *** *p* < 0.001. N: number of nursing homes, RN: registered nurse, LPN: licensed practical nurse, CNA: certified nursing assistant, HCC: hierarchical condition category, FTE: full-time equivalent, HHI: Hirschman–Herfindahl Index.

## Data Availability

Publicly available datasets were analyzed in this study. All analyses were conducted in STATA, version 16.1.

## Supplementary Materials

The following supporting information can be downloaded at: https://www.mdpi.com/article/10.3390/healthcare13040379/s1, Table S1: Variable Definitions and Sources.
