# Supplementary material for: Agency Nursing Staff Utilization and Turnover in Nursing Homes: A Longitudinal Analysis
Source: Healthcare (Basel). 2025 Feb 11;13(4):379. doi: 10.3390/healthcare13040379 (PMC11855161; doi:10.3390/healthcare13040379)
Supplement: Supplementary file 1 [file healthcare-13-00379-s001.zip › healthcare-3360590-supplementary.pdf]

Supplementary Table S1. Variable Definitions and Sources

Variable name	Definitions/Operationalization	Data source
Turnover rate	(Number of nurses who left during the year) / (average number of nurses employed during the year) *(separate for RN, LPN, and CNA)*	Payroll-based journal dataset
Agency nursing staff share	(Agency nursing staff hours) / (Total nursing staff hours) *(separate for RN, LPN, and CNA)*	Payroll-based journal dataset
Size	Total number of certified beds	Provider of Service
Occupancy rate	(Number of residents) / (Number of certified beds)	Provider of Service
Nursing staff hours per resident day	Average number of hours worked by nursing staff per resident day *(separate for RN, LPN, and CNA)*	Payroll-based journal dataset
HCC risk score	Average Hierarchical Condition Category (HCC) risk score is a metric used to estimate the health risk of individuals based on their medical conditions	Post-Acute Care and Hospice Provider Utilization and Payment Public Use Files
Quality star rating	Reflects a facility's performance based on resident health outcomes and care processes, using data such as rehospitalization rates, mobility, and incidence of pressure ulcers, rated on a scale from 1 to 5 stars	Care Compare: Five Star Quality Rating System
Social deprivation index	Composite measure of socioeconomic factors, including poverty, education, single-parent households, housing conditions, and employment	Robert Graham Center
Full-time Nursing staff employment in hospitals	The total number of full-time nursing staff employed in hospitals *(separate for RN, LPN, and CNA)*	Area Health Resources Files
Market competition	Assessed using HHI: The sum of the squares of the market shares (based on total beds) for nursing homes within a county	Area Health Resources Files
MA penetration	The proportion of Medicare Managed Care enrollees over total Medicare enrollees	Area Health Resources Files

Notes: RN: registered nurse, LPN: licensed practical nurse, CNA: certified nursing assistant, HHI: Hirschman-Herfindahl Index, MA: Medicare Advantage
